# Protein informatics combined with multiple data sources enriches the clinical characterization of novel *TRPV4* variant causing an intermediate skeletal dysplasia

**DOI:** 10.1002/mgg3.566

**Published:** 2019-01-28

**Authors:** Stephanie L. Hines, John E. Richter, Ahmed N. Mohammad, Jain Mahim, Paldeep S. Atwal, Thomas R. Caulfield

**Affiliations:** ^1^ Department of Clinical Genomics Mayo Clinic Jacksonville Florida; ^2^ Department of General Internal Medicine Mayo Clinic Jacksonville Florida; ^3^ Center for Individualized Medicine Mayo Clinic Jacksonville Florida; ^4^ Department of Pediatrics Johns Hopkins School of Medicine Baltimore Maryland; ^5^ Atwal Clinic Jacksonville Florida; ^6^ Department of Neuroscience Mayo Clinic Jacksonville Florida; ^7^ Mayo Graduate School, Neurobiology of Disease Mayo Clinic Jacksonville Florida

**Keywords:** Kozlowski type, Maroteaux type, skeletal dysplasia, spondyloepiphyseal dysplasia, spondylometaphyseal dysplasia, transient receptor potential cation channel subfamily V member 4 (TRPV4)

## Abstract

**Background:**

Transient receptor potential cation channel subfamily V member 4 (TRPV4) is an ion channel permeable to Ca^2+^ that is sensitive to physical, hormonal, and chemical stimuli. This protein is expressed in many cell types, including osteoclasts, chondrocytes, and sensory neurons. As such, pathogenic variants of this gene are associated with skeletal dysplasias and neuromuscular disorders. Pathogenesis of these phenotypes is not yet completely understood, but it is known that genotype–phenotype correlations for *TRPV4* pathogenic variants often are not present.

**Methods:**

Newly characterized, suspected pathogenic variant in TRPV4 was analyzed using protein informatics and personalized protein‐level molecular studies, genomic exome analysis, and clinical study.

**Results:**

This statement is demonstrated in the family of our proband, a 47‐year‐old female having the novel c.2401A>G (p.K801E) variant of *TRPV4*. We discuss the common symptoms between the proband, her father, and her daughter, and compare her phenotype to known *TRPV4*‐associated skeletal dysplasias.

**Conclusions:**

Protein informatics and molecular modeling are used to confirm the pathogenicity of the unique *TRPV4* variant found in this family. Multiple data were combined in a comprehensive manner to give complete overall perspective on the patient disease and prognosis.

## 1 INTRODUCTION

*TRPV4* encodes the transient receptor potential cation channel subfamily V member 4 (TRPV4 protein), a gated ion channel which regulates Ca^2+^ uptake and responds to chemical, hormonal, and physical stimuli (OMIM#605427). Pathogenic variants of *TRPV4* can cause both skeletal dysplasias and neuromuscular disorders with these phenotypes not always presenting independently of one another (Schindler et al., 1993). The mechanism by which TRPV4 protein dysfunction causes disease is still debated, although recent literature poses an explanation for the pathogenesis of *TRPV4‐*associated skeletal dysplasias. It has been noted that *TRPV4* has high expression in sensory neurons, osteoclasts, osteoblasts, and chondrocytes (Nishimura et al., 2012). Of particular interest are the chondrocytes, cells that produce cartilage and compose early bones in the process of endochondral ossification. In wild‐type mammalian fetuses, chondrocytes produce a cartilage matrix that is slowly replaced and lengthened by bone, a process that is continued after birth (Mackie, Ahmed, Tatarczuch, Chen, & Mirams, 2008). *TRPV4* pathogenic variants have been experimentally proven to cause incomplete endochondral ossification by interfering with proper chondrocyte function (Leddy et al., 2014; Saitta et al., 2014; Weinstein, Tompson, Chen, Lee, & Cohn, 2014). It is proposed that Ca^2+^ entry through mutant TRPV4 triggers overproduction of follistatin, an inhibitor of bone morphogenetic protein 2 (BMP2). BMP2 is necessary to promote production of several proteins that advance chondrocytes into the hypertrophic stage, a transition that precedes mature bone in endochondral ossification. With an increased follistatin concentration, chondrocytes are less sensitive to BMP2 signaling, meaning that bone is produced more slowly and less consistently than in the wild type (Leddy, McNulty, Guilak, & Liedtke, 2014). This proposed mechanism helps to explain how skeletal dysplasia can result from pathogenic variants of a gene that is expressed in multiple specialized cells.

The six skeletal dysplasias linked to *TRPV4* pathogenic variants vary widely in severity. Familial digital arthropathy‐brachydactyly is the mildest form, presenting only with progressive arthropathy and swelling of the hands and feet starting in early childhood. On the more severe end of the spectrum is metatropic dysplasia, which involves significant pelvic, spinal, and long bone abnormality and results in short‐limb short‐stature dwarfism or premature death (Schindler et al., 1993). Our proband falls somewhere on the spectrum between these extremes presenting with a phenotype that shares aspects of spondyloepiphyseal dysplasia, Maroteaux type, and spondylometaphyseal dysplasia Kozlowski type. Both of these phenotypes are considered *TRPV4*‐associated disorders of intermediate severity. The proband and her daughter present with the novel pathogenic *TRPV4* variant NC_000012.12:c.2401A>G or NC_000012.12:p.Lys801Glu (referred to hereafter as p.K801E) and share some phenotypical features including short digits, abnormal nail beds, and short stature. We present the case of the proband and her family and confirm the pathogenicity of the newly discovered *TRPV4* variant with molecular modeling techniques.

## 2 Case study

**Figure 1:**
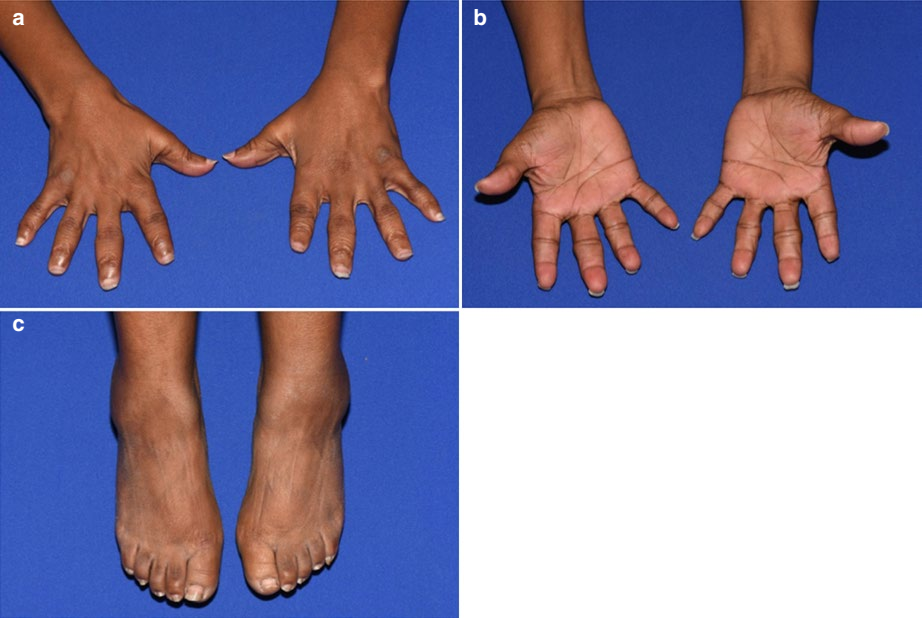
Physical abnormalities in the proband. Multiple digit abnormalities are visualized, including widened PIPs, brachydactyly, and hypoplastic nails. (a) Front of hands. (b) Reverse side of hands. (c) Feet are notable for reduced size, brachydactyly, and hypoplastic nails

**Figure 2:**
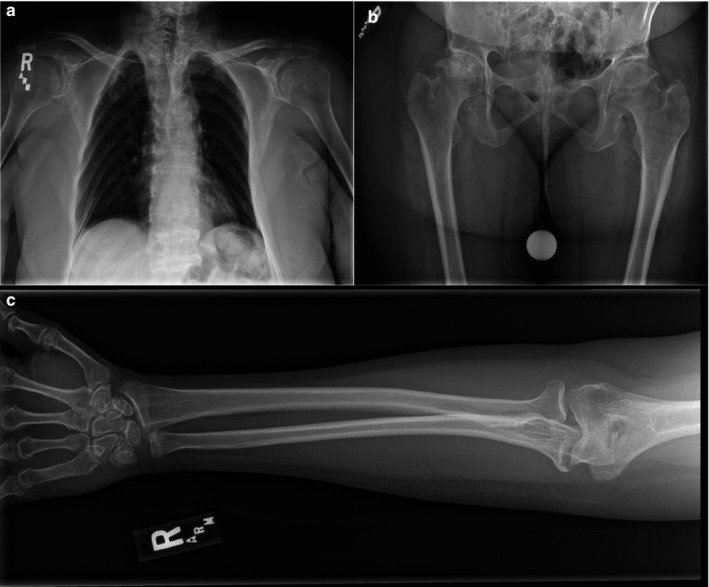
X‐ray images of the proband. (a) Thoracic abnormalities as seen through X‐ray, note platyspondyly, asymmetry of ribs, and absence of scoliosis. (b) X‐ray of the hips, note reduced joint space between hip and right femur. (c) Right arm/hand of proband, metaphyseal abnormality is visualized

**Figure 3:**
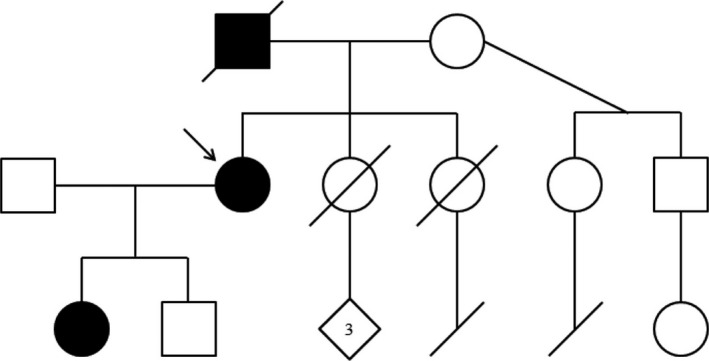
Pedigree of the proband's family. Individuals possessing skeletal abnormalities have been colored black. Note the autosomal‐dominant inheritance pattern—individuals without skeletal abnormalities have children also lacking skeletal abnormality. An arrow marks the proband

Our proband, a 47‐year‐old female, initially presented to our clinic due to a lengthy history of skeletal abnormalities, bone pain, and osteoporosis. She has relatively short stature with a height of 5′2″. She reported that she had normal weight and height at birth with no notable bone abnormalities. As a child, she had scoliosis requiring bracing for correction. Since then, she has experienced “constant” bone and joint pain and her medical records indicated a history of anemia, vitamin D deficiency, and severe osteonecrosis (particularly in her hips). Physical examination of her fingers yielded multiple findings, including widened proximal interphalangeal joints, swan neck deformities, short broad digits, hyperplastic nails, and short nail beds (Figure 1a,b). Her hand joints were hypermobile, her feet were small, and her thorax was abnormally shaped (Figures 1c and 2a). We were also able to confirm her complaints of limited neck and back movement, as she had reported these areas were significant sources of pain. Overall, physical examination suggested a skeletal dysplasia as the source of the proband's symptoms. Skeletal survey and whole exome sequencing + mtDNA analysis were performed to determine the specific disorder. Patients’ pedigree was determined for additional information (Figure 3).

Skeletal X‐rays found multiple abnormalities in the proband, most notably in her spine, pelvis, and long bones. The proband's spine was notable for flattened vertebral bodies, narrowed disk spaces, and end plate sclerosis (Figure 2a). Her pelvis X‐ray was abnormal (Figure 2b). Her long bones exhibited metaphyseal abnormalities. The survey also provided skeletal confirmation for the shortened digits that had been apparent in the proband's physical examination. Additionally, the proband seemed to have diffuse arthropathy of the spine and large/small joints.

The proband's mtDNA analysis was negative, but whole exome sequencing uncovered a novel, likely pathogenic variant that explained her condition. The *TRPV4* variant c.2401A>G (p.K801E) was found in a heterozygous state, consistent with the known autosomal‐dominant transmission of *TRPV4*‐associated disorders (Schindler et al., 1993). It is suspected that the proband received this variant from her father, who died at age 33. Per report, he had abnormal nails, abnormal hips, and short digits. The proband, in turn, passed this variant to her 22‐year‐old daughter, who has known phenotypical features include surgically corrected S‐shaped scoliosis, brachydactyly, macrocephaly, and short stature at 4′11″. The differences in phenotype seen between these family members are unsurprising, as *TRPV4* pathogenic variants do not exhibit clear genotype–phenotype correlations (Nishimura et al., 2012).

Given the skeletal and genetic findings in the proband, she ultimately fell on the disease spectrum somewhere between Maroteaux type and Kozlowski type spondyloepiphyseal dysplasia. At this point in time, her hip pain had increased enough to warrant surgical intervention, which was eventually pursued in spite of her significant osteoporosis. A right total hip arthroplasty was performed, and as of current date has not been complicated by fractures or new osseous lesions. The proband reported a significant improvement as compared to her preoperative level of pain and continues to follow with the clinic to monitor any further degenerative skeletal changes.

## 3 METHODS

### 3.1 Molecular modeling

The Transient receptor potential cation channel subfamily V member 4 (Trpv4 or TRPV4), called TRPV4 gene, is a nonselective calcium cation channel that regulates osmotic sensitivity and mechanical sensitivity in humans. The channel is regulated via a calmodulin‐dependent mechanism with a negative feedback loop. It also plays a function in nonselective cation channel activation induced by 4‐alpha‐phorbol 12, 13‐didecanoate as well as hypotonic stimulation in synoviocytes and also regulates production of IL‐8 (Garcia‐Elias, Lorenzo, Vicente, & Valverde, 2008; Strotmann, Schultz, & Plant, 2003). Lastly, the channel is inhibited through binding with phosphatidylinositol‐4, 5‐bisphosphate (Itoh et al., 2009).

*TRPV4* was taken from the NCBI Reference Accession Sequence: NP_067638: version NP_067638.3, which is encoded for the amino acid sequence: MADSSEGPRAGPGEVAELPGDESGTPGGEAFPLSSLANLFEGEDGSLSPSPADASRPAGP GDGRPNLRMKFQGAFRKGVPNPIDLLESTLYESSVVPGPKKAPMDSLFDYGTYRHHSSD NKRWRKKIIEKQPQSPKAPAPQPPPILKVFNRPILFDIVSRGSTADLDGLLPFLLTHKKRLT DEEFREPSTGKTCLPKALLNLSNGRNDTIPVLLDIAERTGNMREFINSPFRDIYYRGQTAL HIAIERRCKHYVELLVAQGADVHAQARGRFFQPKDEGGYFYFGELPLSLAACTNQPHIV NYLTENPHKKADMRRQDSRGNTVLHALVAIADNTRENTKFVTKMYDLLLLKCARLFP DSNLEAVLNNDGLSPLMMAAKTGKIGIFQHIIRREVTDEDTRHLSRKFKDWAYGPVYSS LYDLSSLDTCGEEASVLEILVYNSKIENRHEMLAVEPINELLRDKWRKFGAVSFYINVVS YLCAMVIFTLTAYYQPLEGTPPYPYRTTVDYLRLAGEVITLFTGVLFFFTNIKDLFMKKC PGVNSLFIDGSFQLLYFIYSVLVIVSAALYLAGIEAYLAVMVFALVLGWMNALYFTRGL KLTGTYSIMIQKILFKDLFRFLLVYLLFMIGYASALVSLLNPCANMKVCNEDQTNCTVPT YPSCRDSETFSTFLLDLFKLTIGMGDLEMLSSTKYPVVFIILLVTYIILTFVLLLNMLIALM GETVGQVSKESKHIWKLQWATTILDIERSFPVFLRKAFRSGEMVTVGKSSDGTPDRRWC FRVDEVNWSHWNQNLGIINEDPGKNETYQYYGFSHTVGRLRRDRWSSVVPRVVELNK NSNPDEVVVPLDSMGNPRCDGHQQGYPRKWRTDDAPL, where the variant of unknown significance (VUS) is p.K801E. The NP_067638.3 sequence was used for computer‐assisted modeling to evaluate the differences between the various X‐ray structures for TRPV4 protein and the composite full‐length (no omissions in structure) versus the VUS. Monte Carlo simulations were performed on the mutant to allow local regional changes for full‐length 871 amino acids and when the p.K801E variant was introduced.

Using Monte Carlo, we were able to refine the multitude of X‐ray structures (all partially missing fragments, side chains, loops, or rotamers), to generate a composite full‐length model for studies with simulations using our previous work with the Yasara SSP/PSSM method (Altschul et al., 1997; Hooft, Sander, Scharf, & Vriend, 1996; Hooft, Vriend, Sander, & Abola, 1996; King & Sternberg, 1996; Krieger et al., 2009; Qiu & Elber, 2006). The structure was relaxed to the YASARA/Amber force field using knowledge‐based potentials within Yasara. The side chains and rotamers were adjusted with knowledge‐based potentials, simulated annealing with explicit solvent, and small equilibration simulations using multiple parallel Yasara refinement protocols (Laskowski, Macarthur, Moss, & Thornton, 1993). Both the entire full‐length structure and the VUS structure were modeled, filling in any gaps, or unresolved portions from the X‐ray.

Refinement of the finalized model using either Schrodinger's LC‐MOD Monte Carlo‐based module or NAMD2 protocols was completed. All refinements started with YASARA generated initial refinement for variant p.K801E or the wild‐type TRPV4 protein (Altschul et al., 1997; Hooft, Sander, et al., 1996; Hooft, Vriend, et al., 1996; Krieger et al., 2009). The superposition and subsequent refinement of the overlapping regions yield a complete model for protein p.K801E or the wild type. The final structures were subjected to energy optimization with PR conjugate gradient with an R‐dependent dielectric to ensure that the stereochemistry was in strict adherence to the force field.

Atom consistency was checked for all 871 amino acids (11,021 atoms), or for the tetrameric complex (44,084 atoms), of the wild‐type model, and for the variant p.K801E, the model's structure had same number of amino acids but with 11,016 atoms per monomer (44,064 for tetramer), verifying correctness of chain name, dihedrals, angles, torsions, non‐bonds, electrostatics, atom‐typing, and parameters (Supplemental Materials). Each model was exported into user‐ready formats: Maestro (MAE), YASARA (PDB) for easy viewing. Model manipulation was completed with Maestro (Macromodel, version 9.8, Schrodinger, LLC, New York, NY, 2017) or Visual Molecular Dynamics (VMD1.92) (Humphrey, Dalke, & Schulten, 1996).

Monte Carlo dynamics searching was completed on each model for conformational sampling, using methods previously described in the literature (Caulfield, 2011; Caulfield & Devkota, 2012; Caulfield, Devkota, & Rollins, 2011; Caulfield & Medina‐Franco, 2011). Briefly, each TRPV4 or TRPV4 VUS was minimized with relaxed restraints using either Steepest Descent or Conjugate Gradient PR and then allowed to undergo the MC search criteria, as previously demonstrated (Caulfield, 2011; Caulfield & Devkota, 2012; Caulfield & Medina‐Franco, 2011; Caulfield et al., 2011). The primary purpose of MC, in this scenario, is examining any conformational variability that may occur with different mutation, structural change, in or about, the region near to the site and possible effect on partner proteins complementary binding disruption with TRPV4 (Table 1, dysplasias and characteristics).

**Table 1:** Summary of TRPV4 phenotypes and disease

TRPV4 phenotypes	Skeletal	Neuro	Muscular	Respiratory	Onset	Progression	Other	Ref
Avascular necrosis of femoral head, primary, 2	Pelvis: degenerative arthritis in hip joints Limbs: AVN of femoral head bilateral, patchy sclerosis, and cystic changes in femoral head, collapse of femoral head				3rd‐4th decade		Based on 1 family	1
Brachyolmia, type 3	Height: normal birth length, short stature, short trunk in childhood Spine: Gibbus, kyphosis, scoliosis, platyspondyly, short neck, barrel chest Pelvis: short femoral neck Hands: clinodactyly	Spinal cord compression					Eyes: hyperopia	2
Digital arthropathy‐brachydactyly, familial	Hands/feet: progressive brachydactyly of middle/distal phalanges, progressive arthropathy of interphalangeal and metatarsophalangeal/metacarpophalangeal joints, radially deviated phalanges				1st decade		Hands with more severity than feet	3
Hereditary motor and sensory neuropathy, type IIC	Short stature Spine: scoliosis Feet: hammertoes, pes cavus	Peripheral neuropathy, upper and lower limbs, abducens nerve and oculomotor nerve palsy, sensory impairment, areflexia	Shoulder girdle muscle atrophy, distal limb muscle weakness/atrophy, wasting hand muscles, intercostal muscle involvement	Vocal cord paresis, respiratory failure (muscle and diaphragm), obstructive sleep apnea, stridor	Variable		Increased hand weakness with cold, earlier onset correlates with increased severity	4
Metatropic dysplasia	Dwarfism, short limbs Narrow chest, short ribs Spine: short spine, scoliosis, kyphosis, long coccyx, coccygeal tail, anisospondyly, platyspondyly Pelvis: Halberd‐shaped pelvis, hypertrophic trochanters Limbs: flared femurs/humerus, dumbbell‐shaped metaphyses Hands: brachydactyly, joint contractures	Fetal akinesia, peripheral axonal neuropathy		Respiratory failure, Exuberant cartilage in trachea/bronchi				5
Parastremmatic dwarfism	Dwarfism, short neck Spine: kyphosis, scoliosis Limbs: bowing of long bones, legs twisted along the long axis, severe genu valgum Joint contractures				Dwarfism noted in late infancy			6
Scapuloperoneal spinal muscular atrophy	Spine: hyperlordosis, scoliosis, kyphosis Pelvis: hip dysplasia Limbs: asymmetric limb length Hands: small, clinodactyly Feet: clubbed, metatarsus varus	Delayed motor development, wide‐based gait, peripheral motor neuropathy, areflexia, decreased sensation	Scapular muscle weakness/atrophy with rounded shoulders, scapular winging, weak neck flexion, torticollis, muscle atrophy/weakness	Voice hoarseness (laryngeal palsy), stridor, respiratory insufficiency in infancy	Birth or infancy	Nonprogressive or slowly progressive		7
SED, Maroteaux type	Spondyloepiphyseal dysplasia, platyspondyly Limbs: short/stubby hands and feet, genu valgum Pelvis: champagne‐glass pelvic inlet							8
Spinal muscular atrophy, distal, congenital, nonprogressive	Arthrogryposis Spine: lordosis, scoliosis, kyphosis Pelvis: hip contractures Limbs: elbow/knee contractures Feet: clubbed, pes equinovarus, pes planus	Areflexia, hyporeflexia	Muscle weakness, distal, lower limbs, muscle atrophy distal, weakness proximal/pelvic girdle, trunk, fatty atrophy, decreased fetal movement		Prenatal or at birth	Nonprogressive		9
Spondylometaphyseal dysplasia, Kozlowski type	Short‐trunk dwarfism, short neck, delayed skeletal maturation, sphenoid hypoplasia, pectus carinatum Spine: odontoid hypoplasia, platyspondyly, scoliosis, kyphoscoliosis, open staircase vertebral bodies Pelvis: square, short iliac wings, flaring of iliac wings, flat irregular acetabula, coxa vara Limbs: mildly curved, metaphyseal flaring or irregularity, prominent joints Hands: hypoplastic carpal bones Short, stubby hands & feet				Normal at birth, waddling gait noted at 15–20 months			10

*Notes*

1. AVN, 2: Mah, W., Sonkusare, S. K., Wang, T., Azeddine, B., Pupavac, M., Carrot‐Zhang, J., Hong, K., Majewski, J., Harvey, E. J., Russell, L., Chalk, C., Rosenblatt, D. S., Nelson, M. T., Seguin, C. Gain‐of‐function mutation in TRPV4 identified in patients with osteonecrosis of the femoral head. J. Med. Genet. 53: 705–709, 2016. [PubMed: 27330106].

2. Brachyolima: Rock, M. J., Prenen, J., Funari, V. A., Funari, T. L., Merriman, B., Nelson, S. F., Lachman, R. S., Wilcox, W. R., Reyno, S., Quadrelli, R., Vaglio, A., Owsianik, G., Janssens, A., Voets, T., Ikegawa, S., Nagai, T., Rimoin, D. L., Nilius, B., Cohn, D. H. Gain‐of‐function mutations in TRPV4 cause autosomal dominant brachyolmia. Nature Genet. 40: 999–1003, 2008.

3. Digital arthropathy‐brachydactyly, familial: Amor, D. J., Tudball, C., Gardner, R. J. M., Lamande, S. R., Bateman, J. F., Savarirayan, R. Familial digital arthropathy‐brachydactyly. Am. J. Med. Genet. 108: 235–240, 2002.

4. Hereditary motor and sensory neuropathy, type IIC: Aharoni, S., Harlalka, G., Offiah, A., Shuper, A., Crosby, A. H., McEntagart, M. Striking phenotypic variability in familial TRPV4‐axonal neuropathy spectrum disorder. Am. J. Med. Genet. 155A: 3153–3156, 2011. [PubMed: 22065612, related citations]; Chen, D.‐H., Sul, Y., Weiss, M., Hillel, A., Lipe, H., Wolff, J., Matsushita, M., Raskind, W., Bird, T. CMT2C with vocal cord paresis associated with short stature and mutations in the TRPV4 gene. Neurology 75: 1968–1975, 2010. [PubMed: 21115951, images, related citations]; Donaghy, M., Kennett, R. Varying occurrence of vocal cord paralysis in a family with autosomal dominant hereditary motor and sensory neuropathy. J. Neurol. 246: 552–555, 1999. [PubMed: 10463355, related citations]; Dyck, P. J., Litchy, W. J., Minnerath, S., Bird, T. D., Chance, P. F., Schaid, D. J., Aronson, A. E. Hereditary motor and sensory neuropathy with diaphragm and vocal cord paresis. Ann. Neurol. 35: 608–615, 1994. [PubMed: 8179305, related citations]; Klein, C. J., Cunningham, J. M., Atkinson, E. J., Schaid, D. J., Hebbring, S. J., Anderson, S. A., Klein, D. M., Dyck, P. J. B., Litchy, W. J., Thibodeau, S. N., Dyck, P. J. The gene for HMSN2C maps to 12q23‐24: a region of neuromuscular disorders. Neurology 60: 1151–1156, 2003. [PubMed: 12682323, related citations]; Landoure, G., Sullivan, J. M., Johnson, J. O., Munns, C. H., Shi, Y., Diallo, O., Gibbs, J. R., Gaudet, R., Ludlow, C. L., Fischbeck, K. H., Traynor, B. J., Burnett, B. G., Sumner, C. J. Exome sequencing identifies a novel TRPV4 mutation in a CMT2C family. Neurology 79: 192–194, 2012. [PubMed: 22675077, related citations]; Landoure, G., Zdebik, A. A., Martinez, T. L., Burnett, B. G., Stanescu, H. C., Inada, H., Shi, Y., Taye, A. A., Kong, L., Munns, C. H., Choo, S. S., Phelps, C. B., and 8 others. Mutations in TRPV4 cause Charcot‐Marie‐Tooth disease type 2C. Nature Genet. 42: 170–174, 2010. [PubMed: 20037586, images, related citations]; McEntagart, M., Norton, N., Williams, H., Teare, M. D., Dunstan, M., Baker, P., Houlden, H., Reilly, M., Wood, N., Harper, P. S., Futreal, P. A., Williams, N., Rahman, N. Localization of the gene for distal hereditary motor neuronopathy VII (dHMN‐VII) to chromosome 2q14. Am. J. Hum. Genet. 68: 1270–1276, 2001. [PubMed: 11294660, images, related citations]; McEntagart, M. E., Reid, S. L., Irrthum, A., Douglas, J. B., Eyre, K. E. D., Donaghy, M. J., Anderson, N. E., Rahman, N. Confirmation of a hereditary motor and sensory neuropathy IIC locus at chromosome 12q23‐q24. Ann. Neurol. 57: 293–297, 2005. Note: Erratum: Ann. Neurol. 57: 609 only, 2005.

5. Metatropic dysplasia: Boden, S. D., Kaplan, F. S., Fallon, M. D., Ruddy, R., Belik, J., Anday, E., Zackai, E., Ellis, J. Metatropic dwarfism: uncoupling of endochondral and perichondral growth. J. Bone Joint Surg. Am. 69: 174–184, 1987. [PubMed: 3805078,]; Camacho, N., Krakow, D., Johnykutty, S., Katzman, P. J., Pepkowitz, S., Vriens, J., Nilius, B., Boyce, B. F., Cohn, D. H. Dominant TRPV4 mutations in nonlethal and lethal metatropic dysplasia. Am. J. Med. Genet. 152A: 1169–1177, 2010. [PubMed: 20425821]; Dai, J., Kim, O.‐H., Cho, T.‐J., Schmidt‐Rimpler, M., Tonoki, H., Takikawa, K., Haga, N., Miyoshi, K., Kitoh, H., Yoo, W.‐J., Choi, I.‐H., Song, H.‐R., and 23 others. Novel and recurrent TRPV4 mutations and their association with distinct phenotypes within the TRPV4 dysplasia family. J. Med. Genet. 47: 704–709, 2010. [PubMed: 20577006,]; Genevieve, D., Le Merrer, M., Feingold, J., Munnich, A., Maroteaux, P., Cormier‐Daire, V. Revisiting metatropic dysplasia: presentation of a series of 19 novel patients and review of the literature. Am. J. Med. Genet. 146A: 992–996, 2008. [PubMed: 18348257,]; Houston, C. S., Awen, C. F., Kent, H. P. Fatal neonatal dwarfism. J. Canad. Assoc. Radiol. 23: 45–61, 1972.; Kannu, P., Aftimos, S., Mayne, V., Donnan, L., Savarirayan, R. Metatropic dysplasia: clinical Genet. 143A: 2512–2522, 2007. [PubMed: 17879966,]; Kaufmann, E. Untersuchungen ueber die sogenannte foetale Rachitis. (Chondrodystrophia foetalis). Berlin: Georg Reimer (pub.) 1892.; Krakow, D., Vriens, J., Camacho, N., Luong, P., Deixler, H., Funari, T. L., Bacino, C. A., Irons, M. B., Holm, I. A., Sadler, L., Okenfuss, E. B., Janssens, A., Voets, T., Rimoin, D. L., Lachman, R. S., Nilius, B., Cohn, D. H. Mutations in the gene encoding the calcium‐permeable ion channel TRPV4 produce spondylometaphyseal dysplasia, Kozlowski type and metatropic dysplasia. Am. J. Hum. Genet. 84: 307–315, 2009. [PubMed: 19232556]; MacCallum, W. G. Chondrodystrophia foetalis: notes on the pathological changes in four cases. Johns Hopkins Hosp. Bull. 26: 182–185, 1915.; Maroteaux, P., Spranger, J. W., Wiedemann, H.‐R. Der metatropische Zwergwuchs. Arch. Kinderheilk. 173: 211–226, 1966. [PubMed: 4963592,]; Michail, J., Matsoukas, J., Theodorou, S. D., Houliaras, K. Maladie de Morquio (osteochondrodystrophie polyepiphysaire deformante) chez deux freres. Helv. Paediat. Acta 11: 403–413, 1956. [PubMed: 13405333,]; Unger, S., Lausch, E., Stanzial, F., Gillessen‐Kaesbach, G., Stefanova, I., Di Stefano, C. M., Bertini, E., Dionisi‐Vici, C., Nilius, B., Zabel, B., Superti‐Furga, A. Fetal akinesia in metatropic dysplasia: the combined phenotype of chondrodysplasia and neuropathy? Am. J. Med. Genet. 155A: 2860–2864, 2011. [PubMed: 21964829,]

6. Parastremmatic dwarfism: Langer, L. O., Jr., Petersen, D., Spranger, J. W. An unusual bone dysplasia: parastremmatic dwarfism. Am. J. Roentgen. Radium Ther. Nucl. Med. 110: 550–560, 1970. [PubMed: 4992387]; Nishimura, G., Dai, J., Lausch, E., Unger, S., Megarbane, A., Kitoh, H., Kim, O. H., Cho, T.‐J., Bedeschi, F., Benedicenti, F., Mendoza‐Londono, R., Silengo, M., Schmidt‐Rimpler, M., Spranger, J., Zabel, B., Ikegawa, S., Superti‐Furga, A. Spondylo‐epiphyseal dysplasia, Maroteaux type (pseudo‐Morquio syndrome type 2), are parastremmatic dysplasia are caused by TRPV4 mutations. Am. J. Med. Genet. 152A: 1443–1449, 2010. [PubMed: 20503319]; Rask, M. R. Morquio‐Brailsford osteochondrodystrophy and osteogenesis imperfecta: report of a patient with both conditions. J. Bone Joint Surg. Am. 45: 561–570, 1963; Sensenbrenner, J. A., Dorst, J. P., Hungerford, D. S. Parastremmatic dwarfism.In: Bergsma, D. : Skeletal Dysplasias. Amsterdam: Excerpta Medica (pub.) 1974. Pp. 425–429.

7. Scapuloperoneal spinal muscular atrophy: Berciano, J., Baets, J., Gallardo, E., Zimon, M., Garcia, A., Lopez‐Laso, E., Combarros, O., Infante, J., Timmerman, V., Jordanova, A., De Jonghe, P. Reduced penetrance in hereditary motor neuropathy caused by TRPV4 arg269‐to‐cys mutation. J. Neurol. 258: 1413–1421, 2011. [PubMed: 21336783]; DeLong, R., Siddique, T. A large New England kindred with autosomal dominant neurogenic scapuloperoneal amyotrophy with unique features. Arch. Neurol. 49: 905–908, 1992. [PubMed: 1520078,]

8. SED, Maroteaux type: Doman, A. N., Maroteaux, P., Lyne, E. D. Spondyloepiphyseal dysplasia of Maroteaux. J. Bone Joint Surg. Am. 72: 1364–1369, 1990. [PubMed: 2229114]; Megarbane, A., Maroteaux, P., Caillaud, C., Le Merrer, M. Spondyloepimetaphyseal dysplasia of Maroteaux (pseudo‐Morquio type II syndrome): report of a new patient and review of the literature. Am. J. Med. Genet. 125A: 61–66, 2004. [PubMed: 14755468]; Nishimura, G., Kizu, R., Kijima, Y., Sakai, K., Kawaguchi, Y., Kimura, T., Matsushita, I., Shirahama, S., Ikeda, T., Ikegawa, S., Hasegawa, T. Spondyloepiphyseal dysplasia Maroteaux type: report of three patients from two families and exclusion of type II collagen defects. Am. J. Med. Genet. 120A: 498–502, 2003.

9. Spinal muscular atrophy, distal, congenital, nonprogressive: Astrea, G., Brisca, G., Fiorillo, C., Valle, M., Tosetti, M., Bruno, C., Santorelli, F. M., Battini, R. Muscle MRI in TRPV4‐related congenital distal SMA. Neurology 78: 364–365, 2012. [PubMed: 22291064]; Berciano, J., Baets, J., Gallardo, E., Zimon, M., Garcia, A., Lopez‐Laso, E., Combarros, O., Infante, J., Timmerman, V., Jordanova, A., De Jonghe, P. Reduced penetrance in hereditary motor neuropathy caused by TRPV4 arg269‐to‐cys mutation. J. Neurol. 258: 1413–1421, 2011. [PubMed: 21336783]; Echaniz‐Laguna, A., Dubourg, O., Carlier, P., Carlier, R.‐Y., Sabouraud, P., Pereon, Y., Chapon, F., Thauvin‐Robinet, C., Laforet, P., Eymard, B., Latour, P., Stojkovic, T. Phenotypic spectrum and incidence of TRPV4 mutations in patients with inherited axonal neuropathy. Neurology 82: 1919–1926, 2014. [PubMed: 24789864]; Fleury, P., Hageman, G. A dominantly inherited lower motor neuron disorder presenting at birth with associated arthrogryposis. J. Neurol. Neurosurg. Psychiat. 48: 1037–1048, 1985. [PubMed: 4056805]; Reddel, S., Ouvrier, R. A., Nicholson, G., Dierick, I., Irobi, J., Timmerman, V., Ryan, M. M. Autosomal dominant congenital spinal muscular atrophy–a possible developmental deficiency of motor neurones? Neuromusc. Disord. 18: 530–535, 2008. [PubMed: 18579380]; van der Vleuten, A. J. W., van Ravenswaaij‐Arts, C. M. A., Frijns, C. J. M., Smits, A. P. T., Hageman, G., Padberg, G. W., Kremer, H. Localisation of the gene for a dominant congenital spinal muscular atrophy predominantly affecting the lower limbs to chromosome 12q23‐q24. Europ. J. Hum. Genet. 6: 376–382, 1998. [PubMed: 9781046,]

10. Spondylometaphyseal dysplasia, Kozlowski type: Dai, J., Kim, O.‐H., Cho, T.‐J., Schmidt‐Rimpler, M., Tonoki, H., Takikawa, K., Haga, N., Miyoshi, K., Kitoh, H., Yoo, W.‐J., Choi, I.‐H., Song, H.‐R., and 23 others. Novel and recurrent TRPV4 mutations and their association with distinct phenotypes within the TRPV4 dysplasia family. J. Med. Genet. 47: 704–709, 2010. [PubMed: 20577006]; Kozlowski, K., Maroteaux, P., Spranger, J. W. La dysostose spondylo‐metaphysaire. Presse Med. 75: 2769–2774, 1967.

## 4 RESULTS

For WT versus the variant p.K801E, we found the stability of the object from energetic calculations for G indicative of object stability shifts that correspond to worsening of the structural integrity from that of the wild type. In particular, for the tetramer, we find the change in object stability (folding state) and ability to partner (form tetramers). Stability of the object for TRPV4 (all 871 amino acids), as measured by G, is 649.73 kcal/mol*å^2^ (162.43 per monomer), and per residue is 0.186 kcal/aa*mol*Å^2^. Whereas the p.K801E variant has a total stability G of 788.92 kcal/mol*Å^2^, per residue of 0.23 kcal/aa*mol*Å^2^ shows an increase of nearly 24% in the per‐residue energy for worsening of stability in the VUS over that of the wild type. If we look instead at direct interaction energy (favorable) for the tetramer complex instead of the individual object stability, we also see an interesting decrease in tetramerization favorability; where the wild‐type, full‐length tetramer has an G (interaction) of −120.13 kcal/mol*Å^2^ that is constructive, versus the p.K801E variant, which only has −59.81 kcal/mol*Å^2^ indicating less robust inter‐monomer binding. We have used this method in the past hypothesis generation and mutagenesis experiments (Caulfield, 2011; Caulfield & Devkota, 2012; Caulfield & Medina‐Franco, 2011; López‐Vallejo et al., 2011; Reumers et al., 2005; Schymkowitz et al., 2005; Zhang et al., 2013).

**Figure 4:**
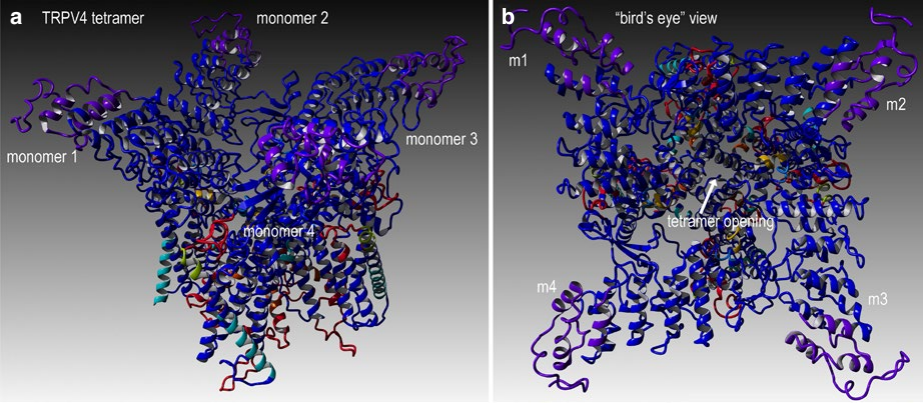
TRPV4 wild‐type structure for full‐length human sequence consisting of 871 amino acids. (a) Full‐length model for the entire TRPV4 structure that shows four monomer proteins that comprise the cation channel, as depicted in ribbons. Colors indicate the composite technique for our hybrid model technology to generate highly reliable structural models for VUS determination. This structural model is complete full‐length sequence including residues and loops missing from X‐ray structures. (b) Rotated view to look down into the channel pore, called the “bird's eye” view. The labeling for both models is indicated. Residues shown are rendered in either “licorice sticks” or van Der Waals (VdW) and using standard element coloring (O‐red, N‐blue, H‐white, S‐yellow) except for the carbon atoms that are colored to match the ribbon color

**Figure 5:**
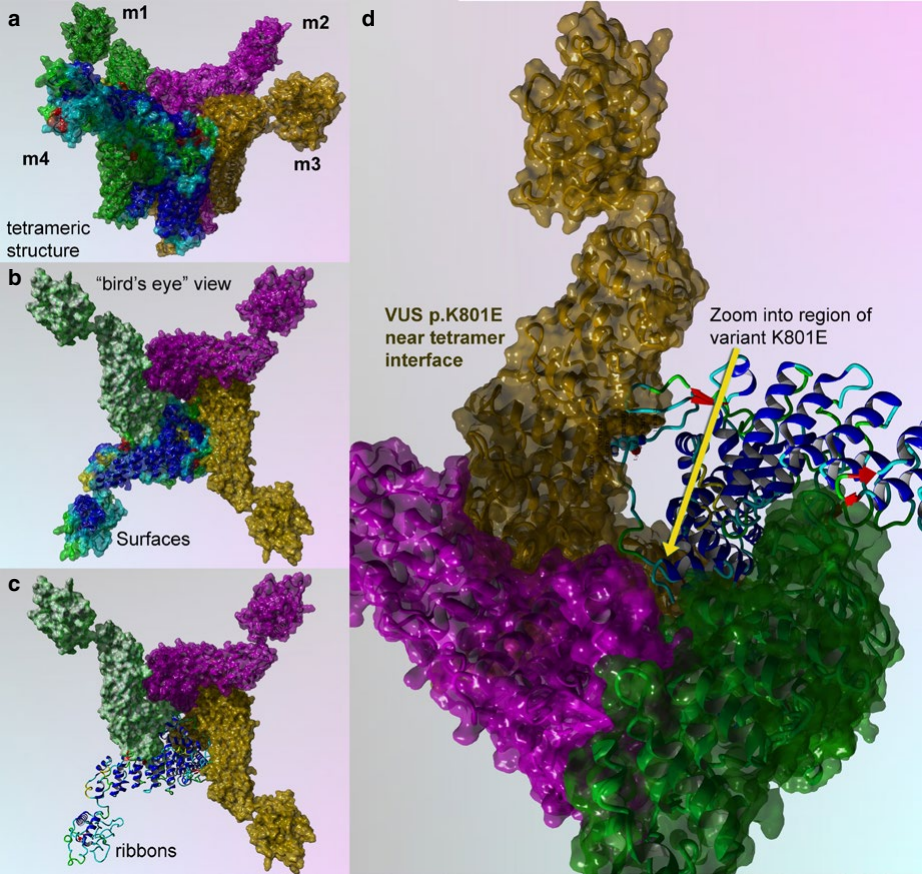
TRPV4 surface and mapping for interaction for wild type and p.K801E variant. (a) Full‐length model for the entire TRPV4 tetrameric structure colored to distinguish the four protein monomer chains. Monomers 1–3 are colored uniformly, and monomer 4 is colored by secondary structure. (b) Bird's eye view for the wild‐type surface rendered model is shown. (c) Key region to zoom into is shown in ribbons and colored by secondary structure. (d) Zoom by 5X and rotation by the X‐Y plane at 135° are done to show position of the VUS (p.K801E), which has a critical point located near the center of the tetramer interface

**Figure 6:**
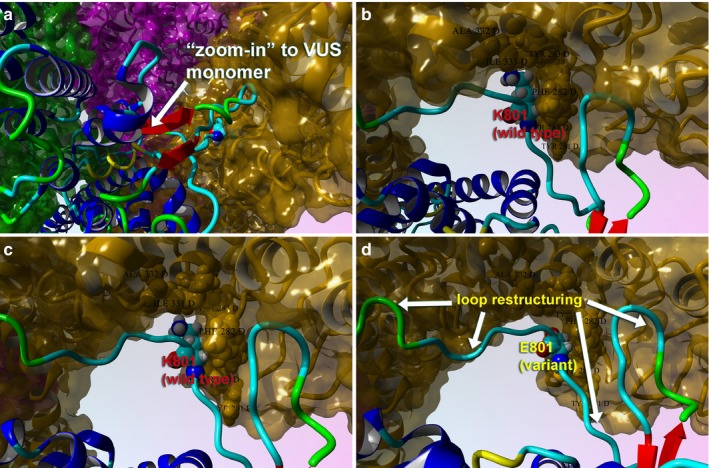
Close‐up views of the TRPV4 variant (p.K801E) surface and variant effect on loop structure and effect on tetrameric interface. (a) Full‐length model for the variant TRPV4 tetrameric structure colored by secondary structure and atom type. Interacting residues between the two proteins are shown. (b) The tetrameric structure for wild type (K801) is shown with same scheme. (c) Close‐up view of the loops is shown for the wild type in this monomer–monomer interaction region. (d) Same view for the p.K801E is shown to illustrate the changes in the loop conformation and the effect on tetramer conformation. This VUS causes the pore structure to be altered for the cation channel, which also demonstrates changes in the protein Gibbs‐free energy for stability

This object stability designates changes in structure deleterious to configuration of the complex from proximate inspection (Figures 4 and 5). Additionally, we examined the local residues that may be useful to explain any change in function (Figure 6). The molecular model for the full structure and its variant form were analyzed using our established techniques (Abdul‐Hay et al., 2013; Ando et al., 2017; Caulfield, 2011; Caulfield & Devkota, 2012; Caulfield, Fiesel, & Springer, 2015; Caulfield & Medina‐Franco, 2011; Caulfield et al., 2011, 2014; Fiesel, Ando, et al., 2015; Fiesel, Caufield, et al., 2015; López‐Vallejo et al., 2011; Puschmann et al., 2017; Zhang et al., 2013). The region around K801 is critical for various loops essential to the interface of the tetramer. Local residues within the 12Å cutoff near the variant site are critical to formation of inter‐monomer interactions that form the pore complex for this cation channel protein, (Figures 4 and 5a,b). Additionally, we can see from the mapping of the surface, plus the loop changes in structure, and resulting effect on the tetramer stability, that the predicted tetramer of TRPV4 for the variant has a substantial loss of the interface compared to the wild type to the p.K801E variant and also the molecular surface shown in the zoomed‐into insets in the figure panel give revealing loss of pore structure that holds the complex (Figures 5 and 6).

The p.K801E tetramer still forms a complex but is not as tightly held together and the pore becomes more disordered and has less regular structure than the wild‐type protein (Figure 5c), which accounts for lowering its interaction potential with partner proteins and the loss of attractive G (Caulfield, 2011; Caulfield & Devkota, 2012; Caulfield & Medina‐Franco, 2011; López‐Vallejo et al., 2011; Reumers et al., 2005; Schymkowitz et al., 2005; Zhang et al., 2013). Further use of protein‐level knowledge as a diagnostic tool for clinician continues to provide new insights and help aid in the development of a database (Caulfield et al., 2018; Harris et al., 2018; Richter et al., 2018; von Roemeling et al., 2018).

## 5 DISCUSSION

The expression of *TPRV4* in a multitude of specialized bone cells is the reason why such a large number of symptoms can result from a single mutation. Overproduction of follistatin, which interferes with endochondral ossification and is thought to cause skeletal dysplasia, only explains part of the proband's phenotype (Leddy et al., 2014). She initially presented with bone pain and osteoporosis/osteonecrosis in addition to her skeletal abnormalities; her skeletal examination revealed widespread arthropathy as a source of her “constant” joint pain. Existing literature on TRPV4′s purpose in osteoclasts and cartilage helps explain these symptoms (Table 1).

Osteoclast differentiation is controlled by a Ca^2+^ signaling pathway, where higher intracellular concentrations of the ion ultimately promote osteoclast‐specific gene activity. As a regulator of Ca^2+^ influx, TRPV4 can effectively control the number of osteoclasts produced in mammals. Pathogenic gain‐of‐function variants of *TRPV4* increase Ca^2+^ uptake, which ultimately raises the number of osteoclasts in an individual. This leads to higher levels of bone resorption than production, a resulting reduction in bone density, and even osteoporosis in some individuals (Leddy et al., 2014; Masuyama et al., 2012). Conversely, *TRPV4* deficiency is known to abate bone loss usually caused by mechanical unloading, a phenomenon that occurs in inactive or bedridden individuals (Mizoguchi et al., 2008). This observation would suggest that a loss‐of‐function variant of *TRPV4* is not behind the proband's osteopenia. Taken with the hypothesis that increased uptake of Ca^2+^ is a generator of skeletal dysplasia, we can likely assume that NC_000012.12:c.2401A>G (p.K801E) is a gain‐of‐function variant.

The arthropathy observed with the proband challenges these above observations. Her arthropathy most closely resembles osteoarthritis, a phenotype that is associated with pathogenic variants of *TRPV4* in both experimental mouse models and human cases (Clark, Votta, Kumar, Liedtke, & Guilak, 2010; Lamandé et al., 2011). These results were seen with knockdown of *TRPV4* expression (mice) or loss‐of‐function *TRPV4* pathogenic variants (humans). Conversely, the skeletal dysplasia and osteoporosis also seen in our proband are known to be associated with gain‐of‐function *TRPV4* variants (Leddy et al., 2014; Masuyama et al., 2012). This contrast suggests that further mechanisms maybe implicated in these manifestations as opposed to simply loss or gain of function. Due to this conflict of symptoms, we cannot say with confidence whether this *TRPV4* variant causes loss of function or gain of function in TRPV4 protein.

Despite the potential *TRPV4* variant conflict between the clinical features of the proband, they have all been seen before in *TRPV4*‐associated disorders. We consider the proband to fit most closely with the spondylometaphyseal dysplasia, Kozlowski type phenotype, though there is some overlap with spondyloepiphyseal dysplasia, Maroteaux type. Both disorders include brachydactyly, platyspondyly, and metaphyseal abnormalities in their phenotypes, signs that were all seen in the proband. However, she does not have bowlegs or a waddling gait, features one might expect to see in these disorders (Schindler et al., 1993). Both also implicate some degree of short‐trunk short‐stature dwarfism, and while our proband stands at only 5′2″, she is taller than the expected heights for most patients having either disease. Autosomal dominant brachyolmia, a milder *TRPV4*‐associated disorder, actually has the closest fit for the proband's height, with an expected adult range between 160 and 168 cm (or between 5′3″ and 5′6″) (Nishimura et al., 2012). The premature osteoarthritis seen in our proband is suggestive of spondylometaphyseal dysplasia, Kozlowski type, as is her childhood scoliosis. Conversely, her osteoporosis is known to be a symptom of spondyloepiphyseal dysplasia, Maroteaux type (Nishimura et al., 2012; Schindler et al., 1993). The indications from the X‐ray support the other findings with regard to the bone osteoporosis and dysplasia observed. The symptomatology of our proband cannot be clearly defined by a single *TRPV4*‐associated disorder; it seems that her case is comparable to a mosaic of 2–3 different disease phenotypes across the *TRPV4*‐spectrum.

Both the proband and her daughter tested positive for the c.2401A>G (p.K801E) variant of *TRPV4*, and while we were unable to test her father, it seems all but certain that he gave the proband this variant. The father's short digits, abnormal nails, and hip abnormalities were all phenotypic features seen in the proband; of these features, only short digits were also seen in her daughter. The father was also of mildly short stature, standing at 5′7″. The cases of this man's daughter and granddaughter combined with his phenotype suggest that he too had skeletal dysplasia (Figure 3). If his hip issues were similar to those seen in the proband, it is possible that he had some form of arthropathy as well. When we investigate the confirmed family members with c.2401A>G (p.K801E), there is some overlap and discrepancy in phenotype. Both the proband and her daughter had scoliosis in early life that required correction, though it would appear the daughter's case was more severe as it required surgical action. It would be important for the daughter to undergo osteopenia evaluation, given a history of femur fracture at age 6. Bone and joint pain are exclusive to the proband, though her daughter might develop these symptoms later in life. The daughter also presents with macrocephaly, a feature that while not seen in the proband is not altogether unexpected given the variability of *TRPV4* genotype–phenotype relationships (Nishimura et al., 2012).

## 6 CONCLUSIONS

Our observation that a loss‐of‐function variant of *TRPV4* is not behind the proband's osteopenia is determined and supports the hypothesis that increased uptake of Ca^2+^ is a generator of skeletal dysplasia, which taken together with the protein informatics and modeling, we find it plausibly likely that NC_000012.12:c.2401A>G (p.K801E) is a gain‐of‐function variant of *TRPV4*. The structural models of these two variants concur with previous clinical data. TRPV4 is added into our repository for the database of all protein‐level VUSs for future use to the clinical community to be made available as an online publicly available resource. Here, we demonstrate the fusion of protein informatics and modeling as another tool to complement the difficult task of variant classification.

## CONFLICT OF INTEREST

All authors declare that they have no conflict of interest.

## AUTHOR CONTRIBUTION

All authors above made substantial contributions to the conception or design of the work; or the acquisition, analysis, or interpretation of data for the work and drafting of the work or revising it critically for important intellectual consent and gave final approval of the version to be published and agreed to be accountable for all aspects of the work in ensuring that questions related to the accuracy or integrity of any part of the work are appropriately investigated and resolved.

## CONSENT FOR PUBLICATION AND INFORMED CONSENT

All procedures followed were in accordance with the ethical standards of the responsible committee on human experimentation (institutional and national) and with the Helsinki Declaration of 1975, as revised in 2000 (5). Informed consent was obtained from all patients for being included in the study.

## AVAILABILITY OF DATA AND MATERIALS

Datasets and materials are detailed in manuscript.

## DISCLOSURES

None.
